# siAKR1C3@PPA complex nucleic acid nanoparticles inhibit castration-resistant prostate cancer in vitro

**DOI:** 10.3389/fonc.2022.1069033

**Published:** 2022-12-16

**Authors:** Xiaoli Cui, Zhou Yao, Tianyu Zhao, Jiahui Guo, Jipeng Ding, Siwei Zhang, Zuowen Liang, Zhengren Wei, Alexis Zoa, Yuantong Tian, Jing Li

**Affiliations:** ^1^ Department of Pharmacology, College of Basic Medical Sciences, Jilin University, Changchun, China; ^2^ Department of Andrology, First Hospital of Jilin University, Changchun, Jilin, China; ^3^ Department of Pharmacology, School of Pharmacy, Gannan Medical University, Ganzhou, China

**Keywords:** AKR1C3, castration-resistant prostate cancer, nucleic acid nanoparticles, PAMAM (G5) dendrimers, 4D label-free proteomics

## Abstract

**Introduction:**

AKR1C3, as a crucial androgenic enzyme, implicates the androgen biosynthesis and promoting prostate cancer cell growth *in vitro*. This study provides a new gene therapy strategy for targeting AKR1C3 to treat castration-resistant prostate cancer.

**Methods:**

siAKR1C3@PPA is assembled from PEG3500, PAMAM, Aptamer-PSMA, and siRNA for AKR1C3. We analyzed the relationship between AKR1C3 expression and the survival rate of prostate cancer patients based on the GEPIA online database to perform disease-free survival, and found that AKR1C3 may be an important factor leading to poor prognosis in prostate cancer. Considering AKR1C3 as a therapeutic target for castration-resistant prostate cancer, we constructed a complex nucleic acid nanoparticle, siAKR1C3@PPA to investigate the inhibitory effect on castration-resistant prostate cancer.

**Results:**

Aptamer-PSMA acts as a target to guide siAKR1C3@PPA into PSMA-positive prostate cancer cells and specifically down regulate AKR1C3. Cyclin D1 was decreased as a result of siAKR1C3@PPA treatment. Changes in Cyclin D1 were consistent with decreased expression of AKR1C3 in LNCaP-AKR1C3 cells and 22RV1 cells. Furthermore, in the LNCaP-AKR1C3 group, 1070 proteins were upregulated and 1015 proteins were downregulated compared to the LNCaP group according to quantitative 4D label-free proteomics. We found 42 proteins involved in cell cycle regulation. In a validated experiment, we demonstrated that PCNP and CINP were up-regulated, and TERF2 and TP53 were down-regulated by western blotting.

**Conclusion:**

We concluded that siAKR1C3@PPA may arrest the cell cycle and affect cell proliferation.

## 1 Introduction

Castration-resistant prostate cancer (CRPC) is now the leading cause of death among prostate cancer (PCa) patients worldwide. The first- line treatment for PCa is androgen deprivation therapy (ADT) to disrupt the androgen receptor (AR) signaling. However, tumors continue to recur and progress to the lethal stage of CRPC. In general, two main mechanisms accelerate prostate (PC) progression to CRPC: (I) activation of the androgen receptor (AR) signaling pathway, and AR variant appearance; (ii) PC cells use their own enzymatic systems to synthesize androgen hormone. Therefore, blocking androgen synthesis in PCa cells is an important approach for the treatment of lethal PCa.

Aldo-keto reductase family 1 member C3 (AKR1C3) is a multifunctional enzyme and is one of the most important genes involved in androgen synthesis and proliferation of PCa cells. A growing number of studies have shown that AKR1C3 is a potential target for CRPC therapy. AKR1C3 activates the ERK signaling pathway to promote epithelial-mesenchymal transition and metastasis (1), conferring PCa cells with enzalutamide resistance (2), and is one of the factors contributing to cross-resistance of second-generation antiandrogen drugs (3). However, the currently studied AKR1C3 inhibitors, such as NSAIDs, steroids and their analogs, all lack specificity and inhibit not only the activity of AKR1C3 but also the activities of its family members AKR1C1 and AKR1C2. Therefore, there is an urgent need to develop specific inhibitors against AKR1C3 targets. Based on four siRNA drugs (Patisirna, Givosirna, Lumasirna and Inclisirna) developed by Alnylam (https://www.alnylam.com/) and approved by the FDA and EC (European Commission). RNA interference was selected to inhibit AKR1C3 specifically in the treatment of CRPC

*In vivo*, delivery of siRNA has always been a key issue in nucleic acid drug development and research. At present, there are four main kinds of carriers,including organic nanoparticles, polymer micelles, lipid nanoparticles (LNPs), dendrimers, etc. Inorganic nanoparticles, includes carbon nanotubes, gold nanoparticles, porous silicon nanoparticles, as well as viral vectors and some hybrid nanoparticles. Patisirna successfully achieved clinical transformation of siRNA molecules in the treatment of diseases using the LNP delivery system, Givosiran is the first RNAi drug targeted to the liver using ESC GalNAc delivery technology. However, there is still a lack of a suitable vector that can load siRNA and target prostate cancer. Poly (-amidoamine) (PAMAM) dendrimers are highly symmetric nanopolymer compounds with precise molecular structures and modified functional groups. More studies have reported that modified PAMAM dendrimers can efficiently carry nucleic acids (DNA, RNA, miRNA, dsRNA) through positive adsorption, making them promising vectors for gene delivery (4–7). However, the positive charge of the amine matrix on the surface of PAMAM makes it somewhat cytotoxic (8, 9). Therefore, polyethylene glycol (PEG) is routinely used to modify PAMAM for shielding, and PAMAM coassembles with PEG as an mRNA nanodelivery carrier, which can efficiently deliver and express mRNA encoding tumor antigens. At the same time, the nanocarriers themselves can activate the innate immune receptor signaling of antigen-presenting cells (10). Therefore, different ratios of PAMAM/PEG were explored in this study to achieve low-toxicity and high-efficiency transport of siRNA.

Moreover, how to accurately identify target cells is an important issue. Utilizing the EPR effect by passive targeting alone is very inefficient, so active targeting strategies using peptides, antibodies, small molecules or aptamers are of great advantage. Aptamers, known as “nucleic acid antibodies”, are single-stranded DNA or RNA obtained by numerically enriched ligand phylogeny techniques. Aptamers have unique three-dimensional structures with high specificity and high binding to their targets (11). Compared with traditional antibodies, aptamers have unique advantages such as low immunogenicity and strong tissue penetration. Aptamers have been used in cancer research therapy to directly inhibit the activity of target molecules by binding to target molecules such as growth factors or oncoproteins, or by introducing and delivering chemotherapeutic drugs or siRNAs to cancer cells (12–14). Aptamer-PSMA (prostate-specific membrane antigen) is an intrinsic glycoprotein present in the membrane of prostate epithelial cells and is highly expressed in prostate cancer tissues (15). PSMA expression levels increase with tumor aggressiveness, androgen resistance, tumor metastasis, and recurrence. PSMA has gradually emerged as a new marker for prostate cancer diagnosis and treatment (16, 17). Therefore, taking advantage of the high specific expression of PSMA in PCa cells, an aptamer targeting PSMA (A10-3.2) can achieve efficient delivery of siAKR1C3@PPA.

Here, we constructed siAKR1C3@PPA nanoparticles to study the inhibitory effect on CRPC. siAKR1C3@PPA was assembled from PEG3500, PAMAM, aptamer-PSMA, and siRNA for AKR1C3. Aptamer-PSMA acts as a target to guide siAKR1C3@PPA into PSMA-positive prostate cancer cells. The antiproliferative effects on prostate cancer cells and their underlying mechanisms were explored. This study provides new evidence for targeting AKR1C3 in CRPC gene therapy.

### 1.1 Reagents and antibodies

The sequence of siAKR1C3 was designed as 5’-GGAACUUUCACCAACAGAUTT-3’. The aptamer-PSMA (Apt-PSMA) sequence was 5’-GGGAGGACGAUGCGGAUCAGCCAUGUUUACGUCACUCCU-(CH_2_)_6_-S−S-(CH_2_)_6_-OH-3’ with 2’-fluoro pyrimidines (18). Aptamer and siRNA were synthesized by Gene Pharma. The poly (amidoamine) (PAMAM) dendrimer G5 and DTT were purchased from Sigma. Heterobifunctional PEG (a-malemidyl-u-N-hydroxysuccinimidyl polyethylene glycol, MAL-PEG-NHS, MW 3500) was purchased from Jen Kem Technology Co., Ltd. (Beijing, China). AKR1C3 1:1000 (rabbit mAb; ab203834; Abcam, Cambridge, UK); β-actin 1:1000 (rabbit mAb; ab203834; Abcam, Cambridge, UK); cyclin D1 1:1000 (rabbit mAb; ab203834; Abcam, Cambridge, UK).

### 1.2 Synthesis of siAKR1C3@PPA and characterization

First, 5.0-generation PAMAM was used to remove methanol solvent with a rotary evaporator to reduce toxicity. The purified PAMAM was prepared in 1 g/mL storage solution with RNase-free water. PAMAM and MAL-PEG-NHS were added to PBS (pH=8.0) at mole ratios of 1:1, 1:1.25, 1:1.5, 1:1.75 and 1:2.0. The mixture, PAMAM-PEG, was protected from light and incubated for 20 minutes at room temperature. The mixture was purified using a centrifugal filtration device (10 k Da molecular weight cutoff; Millipore) and centrifuged for 20 min to remove unreacted PEG (5000 g, 4°C). The buffer was exchanged into phosphate-buffered saline (pH=7.0). The final products (PAMAM-PEG, PP) were then dried in a lyophilizer. Second, Apt-PSMA was dissolved in 0.25 mL 100 M DTT and incubated for 30 min at room temperature without light. Disulfide is activated to form a sulfhydryl group. Apt-PSMA and PAMAM-PEG were conjugated at a molar ratio of 1:2 and incubated at 4°C in the dark for 12 h. The mixture was purified using a centrifugal filtration device (3 k Da molecular weight cutoff; Millipore) and centrifuged for 20 min (5000 g, 4°C). Finally, the purified conjugate of PPA was collected.

PPA and AKR1C3-siRNA were mixed at different ratios of N/P (5, 10, 20, 40, 60, and 80) by vortexing for 30 s. Subsequently, the complexes were incubated for 30 min at room temperature to form the siAKR1C3@PPA by a self-assembly method.

Nuclear magnetic resonance (NMR) spectra of PP were recorded on a NMR spectrometer (Bruker 300, Switzerland). The shape and appearance of siAKR1C3@PPA was confirmed under a transmission electron microscope (JEM-1230, JEOL, Japan). A size and zeta potential NanoZetasizer (NanoZetasizer, Malvern Co., UK) was used to measure the average size and zeta potential of the nanoparticles.

### 1.3 Cell culture and transfection

LNCaP, PC3 and CWR22RV1 cell lines were cultured in RPMI 1640 (HyClone) medium with 10% fetal bovine serum (FBS, GIBCO), penicillin (100 U/mL) and streptomycin ((100 μg/mL). LNCaP-AKR1C3 (LNCaP-AK) cells were generated by transient transfection of LNCaP cells with pCMV6 encoding AKR1C3. Before transfection, LNCaP cells were cultured in medium without androgen (charcoal-stripped FBS, Biological Industries, Beit HaEmek, Israel) and phenol red for 24 h. Transfection was performed when the degree of cell fusion reached 40-50%. Before transfection, 10 μM androstanedione was added as substrate. In the absence of androgen but with substrate, transfection vectors pCMV6 and AKR1C3 plasmids for 12 h were treated as follows. Then drugs were administered according to the experimental groups (LNCaP, LNCaP-AK, LNCaP-AK+PPA, LNCaP-AK+siAKR1C3@PPA). All cells were maintained at 37°C in a humidified incubator containing 5% carbon dioxide. Transfections were performed using serum-free Opti-MEM and Neofect™ (or Lipofectamine 2000 reagent) (Invitrogen, Waltham, MA, USA) according to the manufacturer’s protocol.

### 1.4 SRB and EdU assay

The LNCaP, PC3 and 22RV1 cells in the logarithmic growth phase were seeded in 96-well (1.5×10^3^)or 24-wellplates(5×10^3^). After 24 h of incubation, the cells were treated with following different concentrations of PP for 48 h. Cell viability was evaluatedby the sulforhodamine B (SRB) method. EdU is incorporated into the *DNA* of cells undergoing *DNA* replication. EdU immunofluorescence staining was performed using an EdU kit (Biyuntian, China). PPA and siAKR1C3@PPA were coincubated with LNCaP and 22RV1 cells for 48 h. At 2 h prior to termination of the experiment, EdU was added.

### 1.5 Tumor targeting and imaging assay

The LNCaP and PC3 cells in the logarithmic growth phase were harvested and seeded at 5×10^3^ cells/well in 24-well plates. Cy3-labeled aptamer was added to the culture for 4 h at 37°C. Cy3-labeled siRNA was added for 1 or 4 h at 37°C. Images were captured using an OLYMPUS BX53 microscope.

### 1.6 Cell cycle analysis

Cell cycle analysis was performed using a cell cycle and apoptosis analysis kit (C1052, Beyotime, China). LNCaP cells were washed with PBS after siAKR1C3@PPA was added for 48 h. Then, the cells were fixed with 70% cold ethanol at 4 °C overnight. Samples were stained with staining solution (25 μL propidium iodide, 10 μL RNase) and incubated in the dark for 30 min before cell cycle analysis. The distribution of cells in the cell cycle was measured by a flow cytometer (NovoCyte, ACEA Biosciences, USA).

### 1.7 Western blotting

Cells were cultured as described previously. Cells were lysed in RIPA-1640 (Cell Signaling Technology) on ice for 30 min. For all experiments, 20 μg protein was used, and the primary antibodies were incubated overnight at 4 °C, followed by secondary antibody incubation for 2 h (1:5000 dilution) and chemiluminescence detection. Each Western blot was repeated at least twice. The total protein quantity was assessed using a BCA protein assay (BCA Protein Assay Kit; Pierce Thermo Fisher).

### 1.8 AKR1C3 expression in human PCa tissues and survival analysis

GEO data (GSE2443) were used to compare the expression of AKR1C3 in primary prostate cancer (androgen dependent, Gleasons 5-9, n=10) with that in prostate cancer after androgen ablation (AR-independent, n=10). Outliers were eliminated using a Q test. Disease-free survival analysis based on AKR1C3 expression was obtained from the GEPIA (http://gepia.cancer-pku.cn/index.html) database. A total of 490 prostate cancer patients were selected, among which 245 had high AKR1C3 expression and 245 had low AKR1C3 expression. Genes with a log-rank test P<0.05 were considered statistically significant.

### 1.9 Quantitative 4D label-free proteomics

LNCaP-AK samples and LNCaP samples were routinely cultured for 48 h, digested and stored at -80  °C until the samples were ready for proteomic analysis. Lysate buffer solution (8 M urea, 1% protease inhibitor) was added, and the supernatants were collected. The protein concentration was determined using a BCA kit. The supernatant was digested in solution with trypsin. LC-MS/MS analysis was performed using an EasynLC 1200 chromatography system (Thermo Scientific, USA). When the P value < 0.05, proteins that had a differential expression of≥1.5-fold or ≤ 0.67-fold in the LNCaP-AKR1C3 samples compared with the LNCaP samples were considered differentially expressed. Proteomics analysis was conducted by Jing Jie PTM Biolab Co., Ltd.

### 1.10 Statistical analysis

Data are shown as the mean ± SD. All statistical analyses were performed using SPSS 19.0 (SPSS, Chicago, IL, USA) and Prism 6 (Graph Pad, La Jolla, CA, USA). The data were analyzed by Student’s t test when two groups were compared. P<0.05 was considered significant.

## 2 Results

### 2.1 Elevated AKR1C3 is associate with a low survival rate in patients with androgen-independent PCa

**Figure 1:**
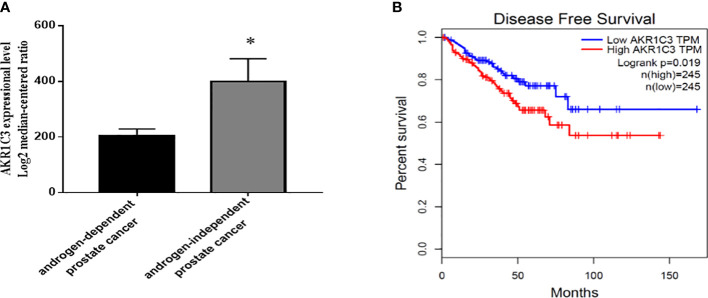
Expression level and survival analysis of AKR1C3 in PCa patients. **(A)** AKR1C3 gene expression analysis using the GEO database. GSE2443: androgen-dependent prostate cancer, n = 8; androgen-independent prostate cancer, n = 8; Data are presented as the mean ± SD; outliers were eliminated using a Q test. **(B)** Disease-free survival among PCa patients with different AKR1C3 expression levels. The plotted data are from the GEPIA database. *P < 0.05.

To explore the potential action of AKR1C3 in the development of PCa, we searched GEO datasets. We found that the expression of AKR1C3 was elevated in androgen-independent microdissected primary tumors compared with androgen-dependent PCa (**Figure 1A**). Disease-free survival analysis (**Figure 1B**) showed that the survival rate of PCa patients with high AKR1C3 expression (red curve) was lower than that of PCa patients with low AKR1C3 expression (blue curve) (P<0.05), AKR1C3 was regarded as a gene for adaptive changes in PCa cells after ADT. It is also an important factor leading to poor prognosis of PCa.

### 2.2 Construction and characterization of nucleic acid nanoparticles siAKR1C3@PPA

**Figure 2:**
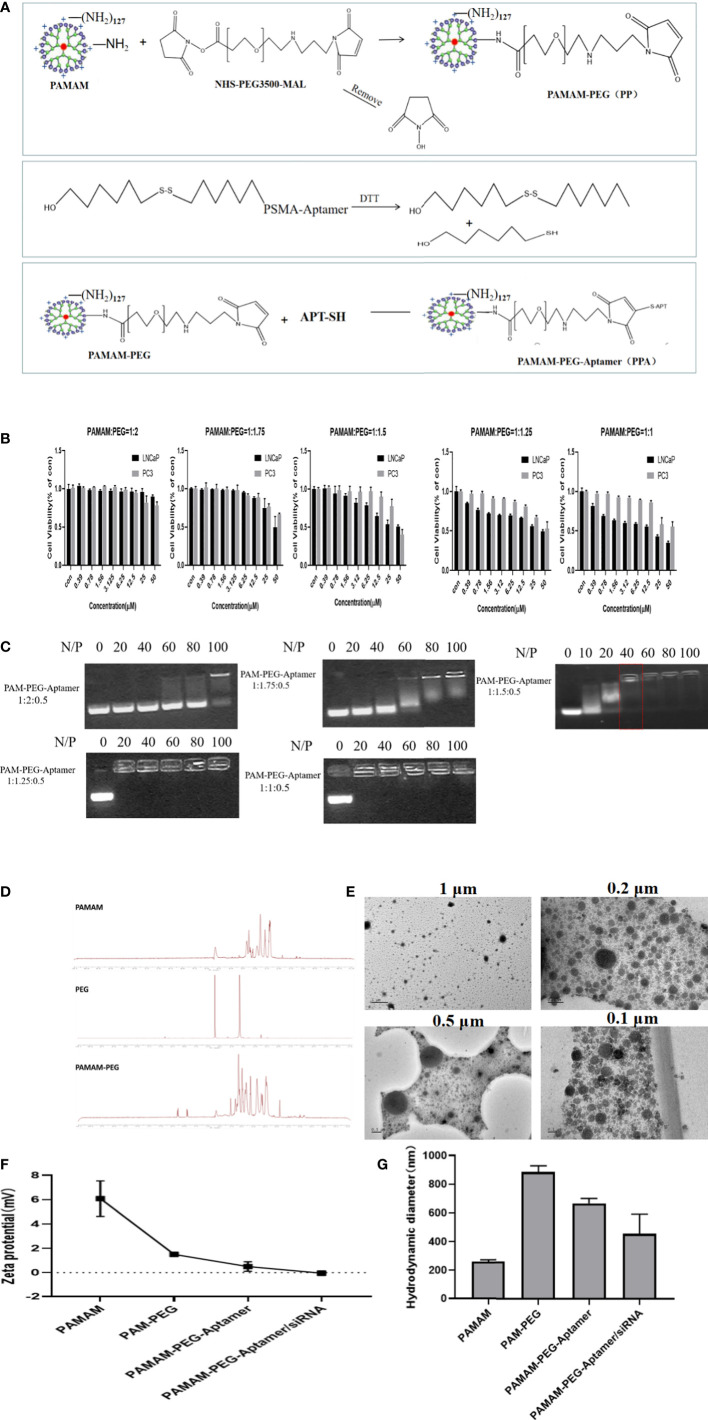
Construction and characterization of complex nucleic acid nanoparticles. **(A)** Schematic Illustration of siAKR1C3@PPA composition and assembly. **(B)** Evaluation of the safety of PP in LNCaP and PC3 cells. Data are presented as the mean ± SD, n=6. **(C)** The optimum ratio of the nucleic acid nanoparticle complex. **(D)** Magnetic spectrum of PAMAM, PEG and PAMAM-PEG. **(E)** Transmission electron microscopy of siAKR1C3@PPA scaled at 1 μM, 0.5 μM, 0.2 μM and 0.1 μM. **(F)** Zeta potential and **(G)** Particle size of nucleic acid nanoparticles. Data are presented as the mean ± SD, n=3.

**Figure 2A** shows the synthesis strategy of siAKR1C3@PPA nanoparticles. The -NH_2_ group of PAMAM was covalently bound to the -NHS group of PEG. The 3’-end of the Apt-PSMA single chain contained a disulfide bond, which was activated to a sulfhydryl group in response to DTT. The sulfhydryl group of Apt-PSMA can continue to react with MAL of PEG. PAMAM and aptamer were respectively attached to dual-function PEG *via* stable covalent bonds, resulting in the PPA that was still positively charged and could self-assemble with siRNA. The resulting nanoparticles are siAKR1C3@PPA.

To explore the best synthesis ratio of PP, we tested the survival of LNCaP and PC3 cells by incubating with different molar ratios of PAMAM: PEG after 48 h. When the PAMAM and PEG molar ratio was 1.5, 1.75 and 2.0, the PAMAM concentration was less than 6.25 μM, and the cell survival rate was above 78%. When the PAMAM/PEG molar ratio was greater than 1.5, PP was relatively safe and could be used for further experiments, as shown in **Figure 2B**. When the ratio of PAMAM to APT was 1:0.5, an appropriate proportion of PEG could effectively bind siRNA. A high proportion of PEG blocked the positive charge of PAMAM and prevented it from binding effectively to siRNA. The results showed that PPA and siRNA could not bind effectively when PAMAM: PEG: Apt-PSMA=1:2:0.5 and 1: 1.75: 0.5. When PAMAM: PEG: Apt-PSMA=1:1.5:0.5, no siRNA bands appeared at N/P=40, indicating that PPA could effectively encapsulate siRNA at N/P =40, as shown in **Figure 2C**. When N/P=40, the PAMAM concentration was 1.398 μM.

PAMAM characteristically peaks at 2.2-3.4 ppm. The NHS characteristic absorption peak was at approximately 3.6 ppm, and the MAL characteristic peak was at 6.7 ppm. The NMR spectrum of PAMAM-PEG had multiple peaks of the repeat units at 3.6 ppm and an MAL characteristic peak at 6.7 ppm. This indicated that -NH_2_ of PAMAM and -NHS of PEG were effectively synthesized, and MAL groups of PEG still existed and could continue to react with Apt-PSMA, as shown in **Figure 2D**. A fifth-generation (G5) PAMAM was spherical in shape, and TEM images (**Figure 2E**) showed that siAKR1C3@PPA was homogeneous, well-dispersed, and spherical in shape.

The zeta potential results (**Figure 2F**) showed that the PAMAM potential was approximately 6 mV, and the potential was reduced to approximately 2 mV after binding with PEG. Both Apt-PSMA and siRNA are negatively charged, which can further reduce the positivity of PP. In the end, the siAKR1C3@PPA Zeta remained positive. Drug nanocarriers are defined as small entities with sizes <500 nm that are easily taken up by tumor cells (19). The results showed that the particle size of siAKR1C3@PPA tended to be 453.9 nm and the polydispersity index (PDI) was 0.29 ± 0.03, as shown in **Figure 2G**.

### 2.3 Apt-PSMA labeling increases the PSMA targeting potential of siAKR1C3@PPA

**Figure 3:**
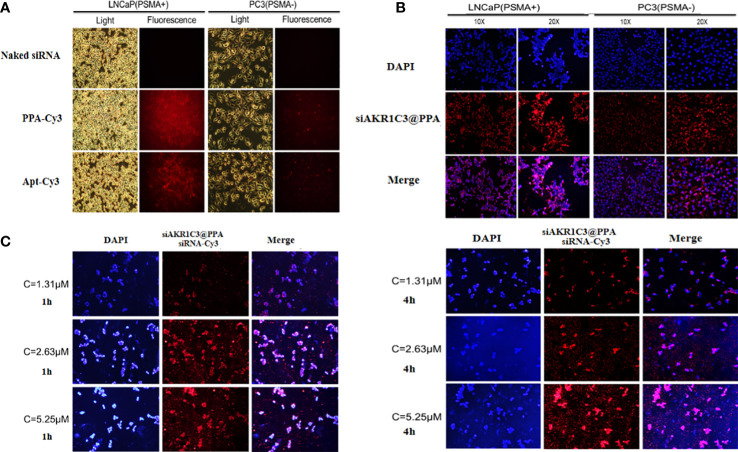
siAKR1C3@PPA can target prostate cancer cells (PSMA^+^). **(A)** Localization of PPA and aptamer in LNCaP and PC3 cells for 4 h at 37°C. Cy3-labeled aptamer. **(B)** Localization of siAKR1C3@PPA in LNCaP and PC3 cells for 4 h at 37°C. Cy3-labeled siRNA. **(C)** The degree of siAKR1C3@PPA internalization is dose- and time dependent. Cy3-labeled siRNA.

To enable the targeting ability of siAKR1C3@PPA, we added an Apt-PSMA component to its formulation. To test the selectivity of siAKR1C3@PPA, we performed an uptake assay in cells with different levels of PSMA. LNCaP cells (PSMA+) and PC3 cells (PSMA-). Cells were treated with either Cy3-labeled PPA or aptamer for 4 h. As shown in **Figure 3A**, in PPA Cy3-treated cells, PSMA+ LNCaP cells showed a high Cy3 signal. However, PC3 cells still show some Cy3 signal, possibly due to PP-mediated cell penetration and basal endocytosis. Apt-PSMA-Cy3-treated cells showed a similar trend, indicating efficient PSMA targeting. To determine whether PPA could successfully deliver siRNA into cells, siRNA was labeled with Cy3. As shown in **Figure 3B**, Cy3-labeled siRNA was translocated into the cytoplasm in LNCaP and PC3 cells. Therefore, PPA can successfully deliver siRNA and has good selectivity for PSMA-positive cells. LNCaP cells were incubated with different concentrations of siAKR1C3@PPA for 1 h or 4 h. The results showed that the fluorescence intensity increased significantly with increasing siAKR1C3@PPA concentration during the same incubation period. At the same concentration of siAKR1C3@PPA, the fluorescence intensity of siRNA-Cy3 increased with the extension of incubation time. Therefore, the content of siRNA carried into cells by PPA was dose- and time dependent, as shown in (**Figure 3C**).

### 2.4 siAKR1C3@PPA inhibits cell proliferation in PCa cells

**Figure 4:**
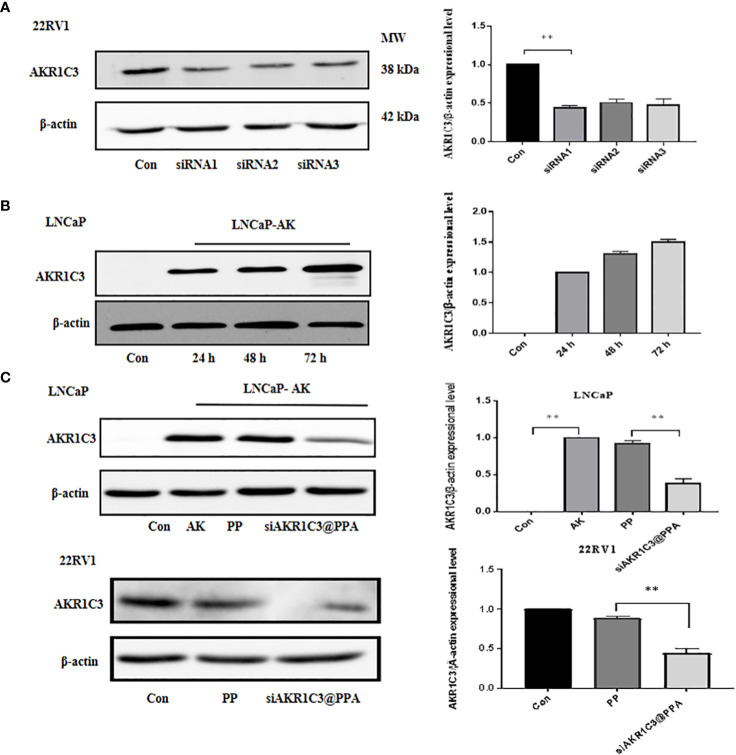
siAKR1C3@PPA transfection decreased AKR1C3 protein expression levels. **(A)** Effectiveness of siAKR1C3 transfection in 22RV1 cells. **(B)** Effectiveness of AKR1C3 plasmid transfection in LNCaP cells. **(C)** siAKR1C3@PPA reduced AKR1C3 protein expression in LNCaP-AKR1C3 and 22RV1 cells. Data are presented as the mean ± SD, n=3; **P < 0.01.

The siRNA1 sequence of AKR1C3 effectively reduced the protein level of AKR1C3 in 22RV1 cells, as shown in **Figure 4A**. To test whether our AKR1C3 siRNA could efficiently reduce AKR1C3 expression, we first overexpressed AKR1C3 in LNCaP cells. As shown in **Figure 4B**, liposome-mediated plasmid delivery was successful, and AKR1C3 levels were maintained for at least 72 h. Then, we loaded these cells with PP or siAKR1C3@PPA. As shown in **Figure 4C**, PP alone did not affect AKR1C3 expression. Upon treatment with siAKR1C3@PPA, AKR1C3 expression was reduced, which was similar in 22RV1 cells with high AKR1C3 expression.

**Figure 5:**
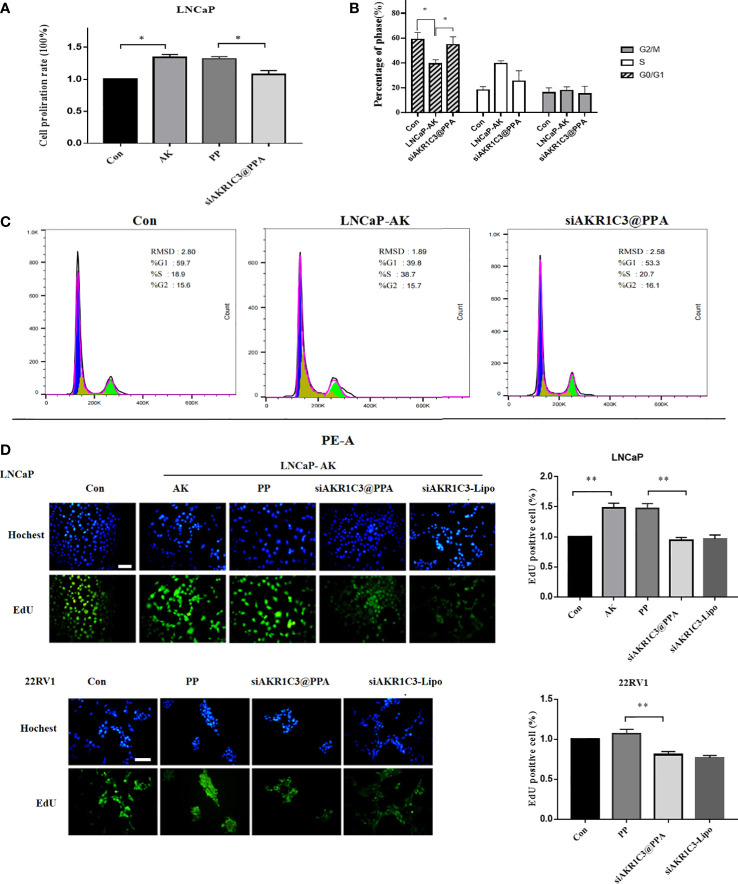
siAKR1C3@PPA inhibited the proliferation of prostate cancer cells. **(A)** Cell proliferation was determined by SRB in LNCaP cells for 48 h Data are presented as the mean ± SD, n=6.**(B, C)** Images and quantified results of the flow cytometric analysis,n=3.**(D)** Cell proliferation was determined by EdU staining (DAPI, blue; EdU-positive cells, green). Bar = 50 μm, n=3. *P < 0.05, **P < 0.01.

Overexpression of AKR1C3 in LNCaP cells effectively promoted cell proliferation (P<0.05, **Figure 5A**), showing that manipulation of AKR1C3 expression could affect PCa cell growth. Flow cytometry showed that AKR1C3 overexpression resulted in a reduction of 19.63% (P<0.05) of cells in the G1 phase compared to the control group. Consistent with its effect on cell growth inhibition, siAKR1C3@PPA also resulted in an increased accumulation of 15.4% of cells in the G1 phase compared to the LNCaP-AK group, as shown in **Figures 5B, C**. These results suggested that siAKR1C3@PPA induced cell cycle arrest in the G1 phase and inhibited LNCaP proliferation.

Next, siAKR1C3@PPA was added to LNCaP-AKR1C3 and 22RV1 cells, and the EdU incorporation results showed that the EdU content decreased. The inhibition rate was 35.98 ± 3.48% in the siAKR1C3@PPA group compared with the PP group in LNCaP-AK cells. In the 22RV1 cell line, compared with the PP group, the inhibition rate was 24.33 ± 2.55% in the siAKR1C3@PPA group, as shown in **Figure 5D**. These data suggest that siAKR1C3@PPA can effectively inhibit the proliferation of PCa cells by affecting DNA synthesis.

### 2.5 siAKR1C3@PPA reduces cyclin D1 expression

**Figure 6:**
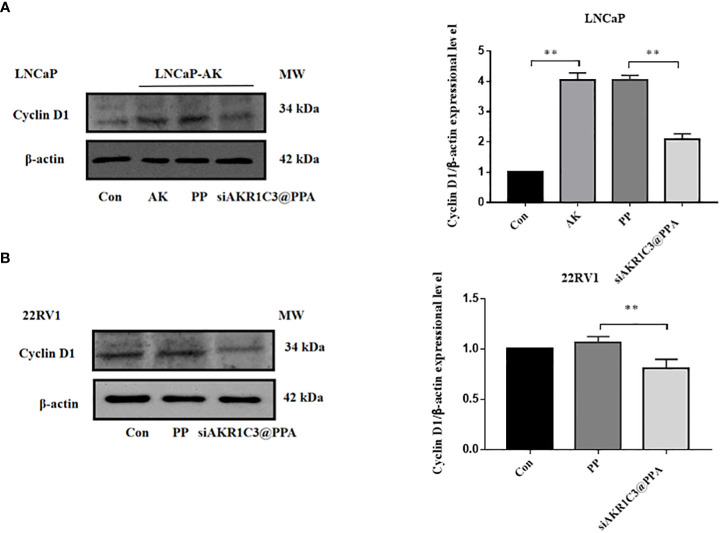
siAKR1C3@PPA affects the expression of cyclinD1 protein in LNCaP. **(A)** and 22RV1 **(B)** cells. Data are presented as the mean ± SD, n=3; **P < 0.01.

Cyclin D1 is one of the many proteins involved in cell cycle regulation, specifically regulating the progression from G1 phase to S phase. We further measured the protein expression level of cyclin D1, and the results showed that the cyclin D1 content in the LNCaP-AKR1C3 group was higher than that in the LNCaP group. The cyclin D1 content in the siAKR1C3@PPA group significantly decreased. The same results were observed in 22RV1 cell lines, as shown in **Figures 6A, B**. We hypothesized that siAKR1C3@PPA might affect cell proliferation by arresting the cell cycle.

### 2.6 Molecular function of AKR1C3 in the LNCaP cell cycle

**Figure 7:**
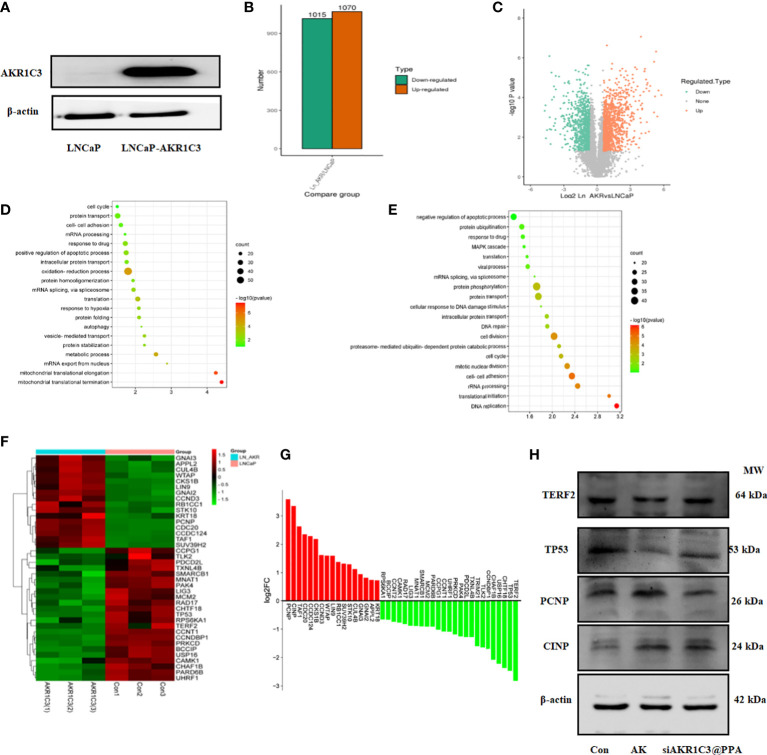
AKR1C3 induces the expression of cycle-related proteins. **(A)** Protein expression of AKR1C3 in LNCaP and LNCaP-AKR1C3 cells. **(B)** Volcano plot of differentially expressed proteins. Orange indicates high relative expression, and green indicates low relative expression. **(C)** Statistical map of differentially expressed proteins. Orange indicates high relative expression, and green indicates low relative expression. **(D)** Biological process (BP) enrichment analysis of upregulated proteins between LNCaP-AKR1C3 cells and LNCaP-vector cells. **(E)** Biological process (BP) enrichment analysis of downregulated proteins in LNCaP- AKR1C3 cells compared with LNCaP cells. **(F)** Heatmap comparing the expression of cell cycle proteins in LNCaP-AKR1C3 cells with that in LNCaP cells. **(G)** The relative fold changes in cycle-related protein levels; values are log2 converted means of relative fold-changes. **(H)** TERF2, TP53, PCNP, and CINP expression was verified by using Western blotting.

To fully clarify the function of AKR1C3 upregulation in PCa, we performed proteomic analysis in LNCaP cells stably expressing AKR1C3 (LNCaP-AKR1C3), as shown in **Figure 7A**. We identified 2085 differentially expressed proteins, among which 1070 proteins were upregulated and 1015 proteins were downregulated, as shown in **Figures 7B, C**. To determine the biological functions of the significantly differentially expressed proteins in LNCaP and LNCaP-AKR1C3 cells, the analysis identified 155 biological process (BP) terms of upregulated proteins and 216 biological process (BP) terms of downregulated proteins (**Supplementary Tables S1**, **S2**). Only the top 20 terms are displayed in **Figures 7D, E**. ‘Cell Cycle’ was the first biological process among the upregulated biological processes. The above results suggested that AKR1C3 may regulate the cell cycle and that downregulation of AKR1C3 may inhibit the proliferation of PCa cells. Therefore, of the 42 proteins involved in cell cycle control, we focused on the functions of the differentially expressed proteins in the cell cycle, as shown in **Figures 7F, G**. These results suggest that AKR1C3 may play an important role in the cell cycle by altering these proteins. As shown in **Figure 7H**, siAKR1C3@PPA downregulated the expression of PCNP and CINP and upregulated the expression of TERF2 and TP53 compared to the LNCaP-AKR1C3 group. Therefore, these results suggest that PPA can package siRNA-AKR1C3 into PCa cells (PSMA-positive) and inhibit their proliferation. AKR1C3 is a promising target in PCa. Our siAKR1C3@PPA strategy efficiently targeted AKR1C3 and benefited PCa.

## 3 Discussion

PCa accounts for more than a quarter of all estimated new cancer cases and the second highest number of estimated deaths in the US (20). Between 27% and 53% of all patients undergoing radical prostatectomy (RP) or radiation therapy (RT) develop a rise in prostate-specific antigen (PSA) levels (PSA recurrence) and progress to CRPC (21). CRPC is an aggressive and incurable disease. The updated guidelines reintroduced both abiraterone combined with prednisone and enzalutamide as first-line therapeutic options for metastatic CRPC (mCRPC) before chemotherapy (21). All patients who receive treatment for mCRPC will eventually progress.

Long-term ADT can induce upregulation of the enzyme required for the synthesis of androgen in PCa cells, and use its own enzyme system to synthesize androgen, so as to promote the reproliferation and metastasis of tumor cells and continue to activate AR signaling. Therefore, inhibiting the synthesis of androgen in prostate tumor cells is one of the strategies for the treatment of CRPC. Abiraterone acetate, a CYP17A1 inhibitor that potently suppresses adrenal and intratumoral steroid biosynthesis, is widely used in the treatment of CRPC. However, treatment with abiraterone is often accompanied by fatigue, peripheral edema, hypertension and other adverse symptoms. Inhibitors of AKR1C3, another intracellular androgen synthesizing enzyme downstream of CYP17A1, protect against these adverse reactions.

AKR1C3 can convert the weak androgen androstanedione to generate testosterone and DHT (22). The mRNA and protein levels of AKR1C3 in CRPC tumors and metastatic tissues were significantly higher than those in normal prostate tissues and carcinoma in situ (23, 24).The high expression of AKR1C3 in tumor tissues is associated with accelerated the progression and metastasis of PCa, and its downregulation can inhibit the growth and invasion of PCa cells. In addition, AKR1C3 overexpression increased the resistance of PCa cells to enzalutamide, abiraterone and chemotherapy drugs, which was reversed by indomethacin, a nonspecific inhibitor of AKR1C3 (22, 25). Thus, AKR1C3, as a key enzyme downstream of the androgen synthesis pathway in tumor cells, can be used as an effective target for the treatment of CRPC. In addition, PGD2 can convert PGH2 to PGD2, and AKR1C3 can further convert PGH2 to 9α, 11β-PGF2 (26). AKR1C3 may also prevent the conversion of PGD2 nonenzyme into PGJ2 and bind to 15-deoxy- Δ12,14-PGJ2(15d-PGJ2) and activate PPARγ to play its antiproliferative role (27).

The negative charge of siRNA is not conducive to endocytosis, whereas the PAMAM dendrimer has an amine surface, which can carry siRNA into the cells. Low generation PAMAM rapidly permeates the entire tumor, reaching the core of the tumor (28). Larger PAMAM dendrimers have lower permeation efficiencies, resulting in distinct intratumoral distributions. The structure of G5 PAMAM becomes globular and densely packed at the periphery. G5 PAMAM, with its spherical structure, was selected as the carrier of siRNA. The higher the generation of PAMAM, the more amino groups on the surface, and the higher the transfection efficiency and cytotoxicity. PEG can shield terminal amino groups from side effects and prolong the circulation time of dendrimers. In our study, PAMAM was combined with aptamer-PSMA by dual functional PEG (MAL-PEG-NHS), and PAMAM carried siRNA to form an efficient active targeting system.

Studies have shown that aptamer-guided targeting of cationic nanocarriers carrying siRNAs is a feasible mean to reduce the off-target effects (29). The expression of PSMA is low in brain, kidney, liver, small intestine and other tissues, and high in many malignant tumors, such as colon cancer, esophageal cancer, thyroid cancer, lung cancer, renal cell cancer and brain tumors, and especially in prostate cancer(http://gepia2.cancer-pku.cn/). 68GA-PSMA-11-PET is the most widely studied and used radioactive tracer in clinical practice based on the PSMA probe (30). When the PSA level is less than or equal to 0.5 ng/ml, the detection rate of patients is approximately 50%. The detection rate was approximately 90% when the PSA level was 3ng/ml (31, 34). Keeping the three-dimensional structures of the aptamer-PSMA complex is the basis for the efficient and specific binding of aptamer A10-3.2 with PSMA. Compared with aptamer A10, aptamer A10-3.2 is easily synthesized with enhanced specificity (35). In this study, a disulfide modified aptamer was used to avoid nuclease degradation and improve its stability. Gorenstein’s research group also confirmed that the disulfide modification increased the binding affinity of by 28-600 times compared with that of the single disulfide aptamer (36).

G1/S processes are closely regulated by cyclin D1, and cyclin-dependent kinase 2 (CDK2) interacts with cyclins, such as cyclin D1, coordinately to control cell cycle progression. To better understand the potential mechanism by which AKR1C3 regulates the cycle of PCa cells, proteomics provides a good research method. CINP and PCNP were the first two upregulated proteins. The Cyclin-dependent kinase 2-interacting protein (CINP) interacts with components of the replication complex and CDK2 and CDC7, thereby providing a functional and physical link between CDK2 and CDC7 during firing of the origins of replication (37). CINP was established as a cofactor of KLF5 crucial for tumor growth in the bladder cancer and HeLa cell lines. Interestingly, the KLF5-CINP interaction also promotes cell proliferation by regulating CyclinD1 (38). Previous reports suggested that the PEST containing nuclear protein (PCNP) was demonstrated to be a tumor suppressor was associated with human cancers, including neuroblastoma, ovarian cancer and lung cancer. PCNP mediates the proliferation, migration, and invasion of human neuroblastoma cells through the MAPK and the PI3K/AKT/mTOR signaling pathways (39). PCNP binding to β-catenin promoted β-catenin nuclear translocation and further activated the Wnt/β-catenin signaling pathway in ovarian cancer (40). PCNP can regulate the progression of human lung adenocarcinoma cells *via* the STAT3/5 and PI3K/Akt/mTOR signaling pathways (41). AKR1C3 may also regulate the P53 signaling pathway in LNCaP cells, which is also a focus of our study in the future.

## 4 Conclusion

**Figure 8:**
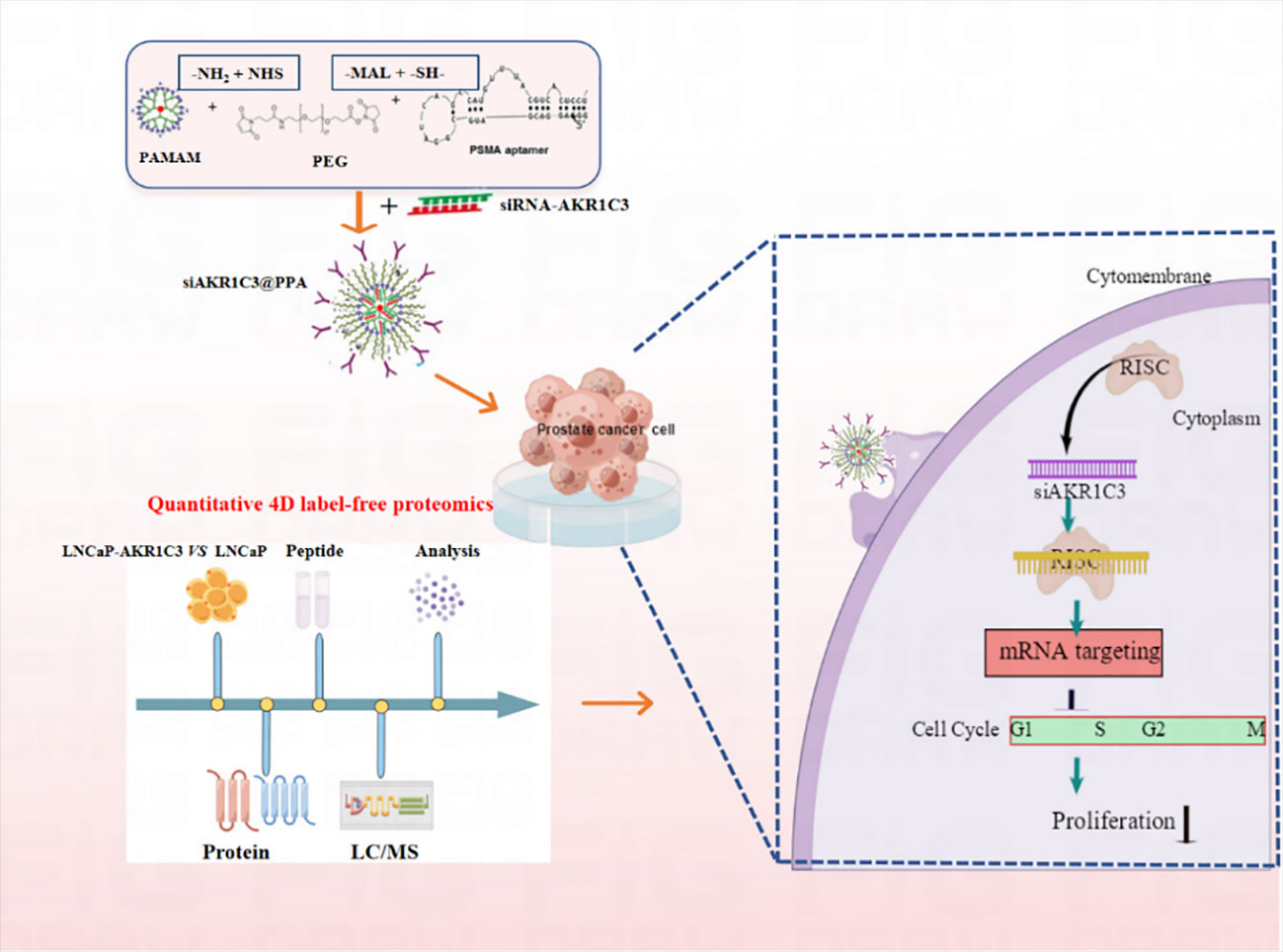
Nucleic acid nanoparticles siAKR1C3@PPA complex targeted inhibits proliferation of CRPC cells. The NH_2_ group of PAMAM was covalently bound to the -NHS group of PEG. The sulfhydryl group of Apt-PSMA can continue to react with MAL of PEG.PAMAM and Aptamer were respectively attached to dual-function PEG via stable covalent bonds, resulting in the PPA that was still positively charged and can self-assemble with siRNA.Aptamer-PSMA acts as a target to guide siAKR1C3@PPA into PSMA-positive prostate cancer cells and specifically down regulate AKR1C3. Regulating Cell Cycle-related protein expression and inhibiting cell proliferation by decreasing AKR1C3 protein expression. Black arrow indicates negative regulation.

We synthesized PPA as a novel carrier for the targeted delivery of siRNA-AKR1C3 to PSMA-positive PCa cells by relying on the targeting ability of Apt-PSMA. The nucleic acid nanoparticle siAKR1C3@PPA complex exhibited excellent targeting capability and inhibited the proliferation of PSMA-positive PCa cells (**Figure 8**). The mechanisms are involved in the suppression of TERF2 and TP53 protein levels and elevated PCNP and CINP. Additionally, through proteomics, we also found other functions of AKR1C3 in LNCaP cells. These data provide many research ideas for further study of AKR1C3 in prostate cancer. However, a limitation of the present study was the lack of *in vivo* experiments; thus, these results require further validation in animal studies.

## Data availability statement

The original contributions presented in the study are included in the article/**Supplementary Material**. Further inquiries can be directed to the corresponding author/s.

## Author contributions

Conceptualization, YT and JL. Methodology, XC. Software, ZY. Validation, XC, TZ and JD. Formal analysis, JG. Investigation, JL. Resources, ZL. Data curation, XC and AZ. Writing—original draft preparation, XC and AZ. Writing—review and editing, SZ. Visualization, ZW. Supervision, JL. Project administration, JL and YT. Funding acquisition, JL and YT. All authors have read and agreed to the published version of the manuscript.
